# Supplementary material for: Effectiveness and Safety of Ayurvedic Medicines in Type 2 Diabetes Mellitus Management: A Systematic Review and Meta-Analysis
Source: Front Pharmacol. 2022 Jun 8;13:821810. doi: 10.3389/fphar.2022.821810 (PMC9213670; doi:10.3389/fphar.2022.821810)
Supplement: Supplementary file 1 [file DataSheet1.zip › Supplementary Material/Forest and Funnel Plots/Cinnamomum aromaticum/Cinnamomum aromaticum.pdf]

Outcomes	Number of studies	Mean Diff. with 95% CI	Heterogeneity (%)
HbA1c	10	-0.16 [-0.45, 0.13]	68.68
FBG	9	1.14 [-6.30, 8.58]	45.71
Fasting insulin*	3	-2.36 [-4.90, 0.18]	83.46
Insulin resistance	1	-0.14 [-0.64, 0.36]	N/A
Body weight	3	-0.52 [-4.05, 3.01]	0.00
BMI	4	-0.42 [-0.90, 0.06]	59.52
Waist circumference	2	-1.44 [-6.10, 3.22]	0.00
SBP	4	-5.36 [-11.97, 1.24]	80.50
DBP	4	-4.18 [-8.26, -0.10]	73.94
Heart rate	1	-0.60 [-6.61, 5.41]	N/A
TC	8	-2.29 [-15.77, 11.20]	84.47
HDL-C	8	-1.78 [-2.37, -1.20]	0.00
LDL-C	7	-2.45 [-9.46, 4.56]	60.26
TG	8	-7.09 [-14.30, 0.12]	0.00

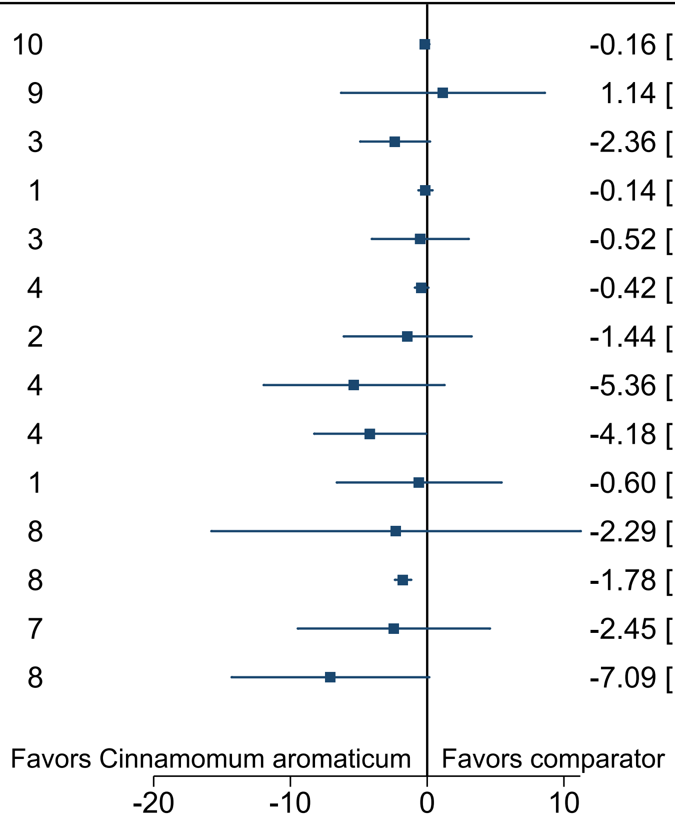

*Favors intervention/comparator as insulin sensitizer
